# The Social Policy Response to COVID-19 – The Failure to Help Vulnerable Children and Elderly People

**DOI:** 10.1007/s11115-021-00560-2

**Published:** 2021-08-31

**Authors:** Tom Christensen

**Affiliations:** Department of Political Science, University of Oslo, Blindern, P.O.box 1097, 0317 Oslo, Norway

**Keywords:** COVID-19, Norway, Social policy responses, Vulnerable groups

## Abstract

Norway’s handling of COVID-19 has been seen as a success. Vulnerable groups among young and elderly people, have however, not been part of this success. Their interests have been defocused in two ways. First, they have not been prioritized in the big picture, because the precautionary principle and health concerns, in particular the capacity questions, have dominated. Second, the more specific social policy measures have been indirect and not particular targeted vulnerable children, youths and old people, and had negative effects for them, such as social isolation and lack of daily support and services, resulting in increasing problems.

## Introduction

COVID-19 has had a profound impact on peoples’ lives around the world, either directly or indirectly. While some of those who have contracted the disease have become seriously ill and died, everyone has had their freedom curtailed and many people have suffered financial hardship as a result of the authorities’ draconian measures (Capano et al., 2020; Hale et al., 2020). Governments around the world have had to deal with at least three simultaneous challenges (Christensen & Lægreid, 2020a). First and foremost, their obligation to protect public health, which has involved taking precautionary measures and making an active effort to curb the spread of the disease and provide adequate healthcare. The second challenge is the economic impact of the virus, which in many cases has received less emphasis in government policy and to some extent been seen as a necessary ‘sacrifice’ in order to save lives (Baldwin & Mauro, 2020). The third challenge is the social and psychological impact, which has been much discussed but received less attention in government policy than the first two concerns. How these various concerns are balanced in official policy and the impact they have depends on a host of structural and cultural factors that influence not only changes and reforms but also crisis management (cf. Pollitt & Bouckaert, 2017).

This article focuses on social policy responses to COVID-19 in Norway. A point of departure is the different aspects of the relationship between social inequality and serious health threats, like pandemics. The pattern of the spread of COVID-19 and its effects on health reflect social inequality within and between countries (Elgar et al., 2020). But our concern here is more with the social policy consequences of COVID-19, meaning how the restrictions and handling of the pandemic have impacted social policy considerations and whether they have targeted certain vulnerable groups. Reports from around the world paint a picture not only of socially disadvantaged groups being harder hit by COVID-19, but also of such groups being put at a disadvantage by the various measures introduced (Chung & Yi, 2021; Martin-Howard and Farmbry, 2020; Yates & Dickinson, 2021). So how will this play out in a country with a relatively high degree of social equality (Roser & Ortiz-Ospina, 2016)?

Crisis management is a rather broad field of study, straddling many disciplines from epidemiology (in the case of COVID-19) to the social sciences. This article examines Norwegian crisis management in terms of public administration and policy, but with a special focus on social policy measures. Two central concepts are important in this strand of literature. The first is *government capacity,* which denotes the resources at a government’s disposal. In the Corona pandemic these are primarily health resources (Boin et al., 2013), i.e., health personnel and equipment, but also economic and social resources. Capacity includes how well prepared a government is for a pandemic, the level of resources used, how well resources are organized, etc. (Boin & van Eeten, 2013). The second concept is that of *government legitimacy,* which concerns the response of the population to the crisis in general and specifically to government measures, which in turn reflects how the government has handled the crisis (Christensen et al., 2016). Have people been supportive and loyally stuck to the rules, or have they been less supportive and protested against the measures? Both reactions have been observable in various countries during the corona crisis. Government capacity and legitimacy exist in a mutually dynamic relationship (Christensen et al., 2016). If government capacity is strong, legitimacy will tend also to be strong, because it gives government leaders leeway to support a lot of different groups, including during a pandemic.

Accordingly, the following questions are asked:

- What have been the priorities in Norway’s social policy response to COVID-19 relative to other major concerns like health and the economy? What has characterized the two basic social policy response measures – income support and job security?
- How has the handling of COVID-19 specifically affected certain vulnerable social groups, such as young and old people, as evaluated by expert reports and reflected in the public debate on this issue? Has the social policy response affected these groups?
- How can one explain the social policy response in general and the effects on the specific vulnerable groups in terms of three perspectives from organization theory – instrumental-structural, cultural-institutional and symbolic?

The article contain a critical analysis of how vulnerable groups, in particular certain groups of children, youths and certain groups of old people, all seen as vulnerable groups, were at a disadvantage both regarding the general regulatory measures and the more specific social policy measures, with overall negative consequences.

The article begins with a short theory section, followed by a brief section describing our methodology. This is followed by an outline of the Norwegian political-administrative context and a review of the corona response in Norway so far. The Norwegian social policy response to COVID-19 is then focused broadly, outlining some of the debates as well as the actual measures taken. The final sections are a discussion and a conclusion in which some of the main features are analyzed and the implications of the social policy response considered.

## Theoretical Basis

Organization theory offers several perspectives that may explain why a government like the Norwegian one would choose certain regulations and measures rather than others to fight COVID-19 and how these measures are arrived at. It also provides a framework for understanding the more specific social policy response to the pandemic (cf. Christensen et al., 2020). An *instrumental-structural perspective* would claim that central political and administrative actors play an important role in the measures chosen to manage a crisis like the corona pandemic (Boin et al., 2005). The perspective comes in two versions (March & Olsen, 1983): a hierarchical one, which claims a political leadership is homogeneous; and a negotiational one, which asserts that the leadership will take a heterogeneous approach to defining and solving problems and that decisions will be the result of negotiations and compromises.

Based on this perspective, the focus is on two aspects of the response: the definition aspect, meaning how goals, problems and solutions are defined; and the activation aspect, meaning who is involved in the various phases of decision-making on social policy (cf. March & Olsen, 1976).

Second, a *cultural-institutional perspective* focuses on how public organizations gradually develop certain unique informal cultural norms and values through a path-dependent process (Krasner, 1988; Selznick, 1957). These cultural traditions will influence later decisions; in other words, the past is overrepresented in the current situation. The term for this is a logic of appropriateness, whereby decision-making is dependent on how the interests and norms currently at stake match the historically evolved path and identity of a public organization (March, 1994).

In this study, it’s discussed whether there are certain more general features of Norwegian political-administrative culture that would explain the response to COVID-19 and, more specifically, whether certain cultural characteristics of the social policy field are relevant here, such as the role of experts.

A third perspective is a *neo-institutional* or *myth/symbolic perspective.* This asserts that organizations have two types of environment: a technical one, which is about instrumental processes; and an institutional one, which alludes to the fact that public leaders often use myths and symbols when making decisions (Meyer & Rowan, 1977). The reason for this is that using symbols gives them more flexibility in balancing instrumental actions (Brunsson, 1989). Using symbols that signal rationality and modernity may increase their public legitimacy.

Viewing the corona response in general and the social policy response in particular through this prism thus involves looking for the typical use of symbols by political-administrative leaders, either to support instrumental action or as a substitute for instrumental action, and more specifically related to social policy (cf. Carpenter, 2010).

It should also be noted that vulnerable and marginalized groups overall are at a disadvantage in many ways. First, they have less social and political resources than other groups, often gathered in the concept of less social capital, with implications for collective action (Ostrom, 1994). Second, they are to a lower degree organized in social groups or interest organizations. Third, they in fact participate relatively less in political processes (Armingeon & Schädel, 2015). All this, taken together, have the effect that vulnerable or marginalized groups are less influencing political process, resulting in decisions on regulations, allocation and measures, and therefore are at a disadvantage in getting services and goods (Rothbart, 2019). They are exposed to the Matthew principle – “For unto every one that hath shall be given, and he shall have abundance; but from him that hath not shall be taken away even that which he hath” (Matthew 13:12). It’s looked into whether this cumulative disadvantage is typical for the COVID-19 social policy responses in Norway.

## Methodology

The data used for this study is a combination of public documents, including reports from expert bodies and groups (Expert group, 2020; Coordination group 1 and 2, 2020), information from the media and secondary data from relevant studies of the pandemic. For the first three months the government gave daily press briefings. These provide a large set of information regarding both regulations and measures, including those relevant to social policy. These briefings were also a good source for analyzing the symbols of crisis communication (Christensen & Lægreid, 2020b). Norwegian public broadcasting channels gave the pandemic about ten hours’ coverage every day for 2–3 months, which also emphasized diverse social concerns presented by experts.

## The Norwegian Context

### General Systemic Features

Norway has a population of only 5.37 million spread across a vast territory. It follows the Nordic welfare state model and is known for its well-functioning democracy and affluence based on its oil reserves and on the Norwegian Sovereign Wealth Fund, which is a pension fund. Norway has a rather centralized political system with many parties and has had minority coalition governments for most of the past three decades. The current coalition consists of the dominant Conservative Party, the Christian Democratic Party and the Liberal Party. The tripartite consensus system, which takes the form of collaboration between the government and employers’ and employees’ organizations, is very important in Norway (Christensen, 2003). This system has resulted in laws and measures that provide for a high level of job security and generous social benefits.

Norway has a universal healthcare system with a lot of resources, giving all citizens access to good healthcare regardless of their social background or personal resources. Hospitals are generally under the control of the state, which allows more control over capacity in a crisis situation. The share of old people in the population is low compared with other European countries, and the educational level is very high.

### Relevant Features of the Welfare System

Norwegian welfare provision is generous and either universal or means-tested. The 2005 reform of the welfare system merged the pensions and employment administrations and co-located them on the local level where they also collaborate with other social services (Lægreid & Rykkja, 2015; Røysum, 2013). The reason for this reform was primarily to better serve the needs of multiple-service users.

The national pension scheme is based on a combination of general taxes and contributions. Employers pay an universal employer’s tax, while employees and self-employed pay social security contributions based on their personal income. The central agency in the welfare administration is called the NAV. It has a vast portfolio of tasks, some of which are specifically relevant for the social policy response to COVID-19. First of all, it deals with people seeking employment or who have been laid off fully or permanently. Second, it is responsible for sick pay and for helping employees who have been off sick return to work. Third, it provides economic support in the transition back to employment, in the form of a benefit called a ‘job-seeker’s allowance’. Third, it provides housing benefit and, where necessary, benefits to cover food and other needs, including economic advice and help with debts. Fourth, it administers various kinds of maternity and child benefit, including that paid to single parents as well as pension benefits and disability allowance.

### Crisis Management Design and the Development of COVID-19

The Ministry of Health and Care Services (MH) is the central crisis management ministry responsible for handling an epidemic or pandemic in Norway, and the main expert bodies are its subordinate agencies: the Norwegian Directorate of Health (NDH) and the Norwegian Institute of Public Health (NIPH) (Christensen & Lægreid, 2020a). The prime minister and the cabinet are also central actors in collaboration with parliament, since the current government is a minority coalition government. The Ministry of Labor and Social Affairs, which is responsible for labor market policy, the working environment, pensions and welfare policy, has not been a central actor during most of the handling of the pandemic (Christensen & Lægreid, 2020b).

The first infected person was registered on February 26, 2020. The geographical spread of the disease in Norway has been very uneven all along, reflecting social status, vacation habits and population density. Oslo, the capital, has had the highest number of cases per capita throughout. The number of infected and hospitalized patients increased rapidly until March 28, but after that remained stable for a while and then decreased substantially until a new surge in late October. This second wave, lasting two to three months, was considerably weaker, but a new, stronger third wave, involving the Alpha mutant, emerged in February 2021; the rate of infection began to slow down again from early April. As of July 9, 2021, a total of 132,885 people had been infected during the pandemic in Norway. 796 deaths had been registered and the average age of the deceased was 82.

The Norwegian government hesitated about what to do until March 12, 2020, when it implemented several draconian regulations (Christensen & Lægreid, 2020a). The most important regulatory measures were similar to those adopted by most other countries; they were introduced during the first months of the outbreak and are largely still in place (NOGOV, 2020a). The restrictions were based on the precautionary principle and gave priority to health over economic and especially social factors. They were also standardized national regulations that took precedence over local flexibility. Overall, the response was a combination of mandatory regulations and softer advice (Christensen & Lægreid, 2020b).

291,000 people, or 10.4% of the labor force, were registered as fully unemployed by March 24, 2020. By comparison, two weeks earlier the unemployment rate had been 2.3%. As of June 8, 2021, the figure was 3.4%, reflecting economic recovery. To mitigate the negative economic effects of the restrictions, the Norwegian government introduced support measures in several steps between early March and early June 2020, and many of these have been extended and prolonged through the second and third waves.

The first were immediate measures to support jobs, help businesses to avoid unnecessary layoffs and prevent bankruptcies in viable companies. Second, guarantees and loans in crisis support for businesses were followed by compensation schemes for workers in the culture, voluntary and sports sectors. Third, additional financial support was approved to sustain businesses that had been severely affected by the measures to contain the pandemic. Fourth, additional measures were directed at businesses that had been particularly hard hit during the pandemic, including cash support for enterprises. Along the way, there were several smaller, but important sector-oriented packages. All together these packages have amounted to about 250 billion NOK.

## Social Policy Response

### Broad Trends

Broadly speaking, the Norwegian private sector has been far more affected by COVID-19 than the public sector, resulting in massive permanent or temporary lay-offs (Christensen & Lægreid, 2020b). This is because job security guarantees for public employees are very strong in Norway. The focus of the social policy response was therefore on the private sector and on different kinds of generous compensation packages, of which about 70 billion NOK were related to NAV and economically oriented measures in this sector. The main purpose of the various public compensation packages presented above was either to compensate businesses directly or to help their employees, while social policy was a secondary focus with a greater emphasis on economic aspects than on the situation of vulnerable groups.

### Individually Oriented Measures

The welfare administration agency, NAV, which is subordinate to the Ministry of Labor and Social Affairs, has been the main body responsible for the individual compensation programs (NAV, 2020). The logic was that ordinary programs should receive a financial boost or be extended by the government, since more people needed them, and that special programs adapted to the COVID-19 situation should be established (NOGOV, 2020b).

The first COVID-19-related compensation program was the *wage-compensation program for temporarily laid-off people* introduced on March 20, 2020; this was for people who were fully or partially laid off temporarily and covered an annual wage of up to 60,000 euros. The benefit is means-tested and takes into account when the employee was laid off, the previous wage level, whether the employer has provided any financial support, the number of days that an employee has worked while temporarily laid off and other benefits received.

After receiving this form of wage compensation for some time, temporarily laid-off employees have the right to *unemployment benefit*. When the program started, unemployment benefit eligibility was for six months, but after a lot of pressure from the opposition in parliament, it was decided that the program should be extended to a year from November 1, 2020, which meant a major additional financial burden on the state. If an employee does not qualify for unemployment benefit, another option is *social assistance* from the welfare agency.

A third new program targeted the *self-employed* and *freelancers* and compensates for their losses. This program was later extended until Sep. 30, 2021. Fourth, the *work assessment allowance program*, established over the last decade in Norway, gives people an income in periods during which they are ill or injured and require assistance from the welfare administration to return to work. One important condition for receiving the allowance is the ‘duty to act’, meaning that the users must actively contribute to the process of returning to work and must follow an activity plan. As a result of the pandemic, the work allowance has been temporarily extended for several groups.

A fifth social policy program is *sickness benefits for self-employed people* and *freelancers,* where eligibility criteria have been relaxed during the pandemic. Sixth, the *care benefit days* program was also changed in response to the pandemic, both for employees and for the self-employed and freelancers, and the number of care days was doubled to forty. The most obvious reason for the need for more care benefit days was school closures, distance learning and parents working from home. Seventh, the *program of economic social assistance* was also made more generous during the pandemic. Obviously, those who lost their jobs or were temporarily laid off required financial assistance, but the effects of the various COVID-19 regulations were also an important reason for extending this program.

Adding to all the individually oriented social policy programs, certain collective measures, administered by the tax authorities, were introduced (Tax, 2020). These included changes in tax rates, fees, rules and deadlines, less coercive fines and more relaxed application of some of the rules. The packages were intended to supplement the individually oriented programs, the idea being that by increasing support for businesses, more of them could remain economically viable and keep their employees, so there would be less of a need for individually oriented social programs.

Overall, the social policy response to the pandemic in Norway shows a mixed pattern. Balancing the different main considerations, the political executive prioritized health, using the precautionary principle, leading to a large and complicated set of measures (Christensen & Lægreid, 2020a). Next came large compensation packages for business and the labor market. The social policy response had the lowest priority. Nevertheless, the tailored social policy measures were generous and there were also positive side-effects from the economic packages for businesses.

### Related Issues and Debates – Vulnerable Groups

One way to focus on the social policy response during the pandemic is to look at the broader picture; another is to look more closely at the most vulnerable groups, i.e., those disadvantaged by a lack of social resources, discrimination based on race, religion, age, disability or location, those in need of care, etc. (Gaynor & Wilson, 2020; Wright & Merritt, 2020) At some point during the first wave, Norwegian politicians talked about vulnerable children and young people, but this was never treated as an important issue. In principle, various services and measures for these groups could have been prioritized during the pandemic, such as keeping kindergartens, schools and special institutions open in order to give them extra services in this institutional framework, but in practice the question of vulnerable groups became deemphasized.

Some aspects of this question were discussed during the first five to six weeks after the imposition of measures on March 12, when kindergartens and schools in Norway were closed; it resurfaced during the second and third waves likewise in connection with partially closed or shorter hours in some kindergartens and schools (Hafstad et al., 2021; Harris & Sandal, 2021; Øverlien, 2020; Ursin et al., 2020):

- The family situation overall became more difficult for children and young people, because of family members having to work from home, and children’s education and development suffered.
- In dysfunctional families, one effect was potentially more exposure of children to abuse from parents and relatives, since vulnerable children were no longer receiving daily support in kindergartens, schools and special institutions and the previous monitoring function of these institutions disappeared.
- Another potential effect of closures and infection-curbing measures was more social isolation in the absence of the daily school routines and contact with friends outside school.
- The lack of daily teaching, especially in challenged families, had a negative aspect on children’s social skills. Vulnerable children were particularly hard hit here, because resources were not available to compensate for the crisis situation.
- In addition, programs for children and young people in special institutions had most of these activities suspended because of the danger of infection.

After some public pressure, the government decided in late March 2020 to establish a small group of experts to discuss and report on the school-related measures and their effects during the pandemic. In its report about both the consequences of the pandemic and potential measures, the group focused primarily on vulnerable children and young people, who were said to encompass about 20% of this section of the population (Expert group, 2020). The report pointed out that vulnerable children and young people normally needed extra measures and were often multiple-service users, and that the pandemic had worsened their situation overall. It mentioned children and young people with various disabilities and those affected by substance abuse, violence in the family, and mental health issues like depression, etc. (Kim and Jung, 2020).

More specifically, the report mentioned that 3% of all children in kindergartens in Norway had received so-called ‘individual decisions’ related to special pedagogical measures proposed after an external expert evaluation of their special needs by a psychological-pedagogical institution. The report also pointed out that the closure of schools occasioned by the crisis made it more difficult to judge the potential problems of children at home and that families often failed to seek help because of fears of stigmatization. Some vulnerable children had previously been placed in kindergartens as a result of measures taken by the child care services so that closing kindergartens often resulted in increased care failure.

The report pointed out that schools in Norway had a wide range of students, many of whom had special needs, such as adapted teaching and language training (mainly for immigrants). In 2019, this encompassed 7.7% and 6.4%, respectively, of all students in elementary and middle schools, while the percentage was lower in high school. Overall, 20% of all school students have some kind of developmental and/or learning difficulty. The report concluded that while schools were closed it was impossible to cover these needs in any systematic way. Because of the pandemic, many institutions or services were closed, resulting in a lack of resources and in priority problems.^1^

The report was short on specific measures designed to ease the problems identified. Its recommendations focused mainly on urging the authorities to re-open the kindergartens and schools quickly and on detailing how to do this. However, political leaders decided to wait another three to four weeks before re-opening kindergartens and schools, so that the problems continued for vulnerable groups. In the second and third waves, the measures related to kindergartens and schools were generally stricter.

However, a broader coordination group was established with representatives from all the affected public institutions – eight in all, mainly agencies. It was to report to the political leaders every second week from April 20. In its first report (Coordination group 1, 2020), the group discussed why the services for vulnerable children and young people had been scaled back during the pandemic. One reason given was the various infection regulations; another was staff shortages caused by sickness and quarantine; and a third, reluctance by the affected individuals and their families to seek help. One major problem was that many of the relevant social institutions for the vulnerable groups, like family and child protection institutions and health institutions, had been totally or partially closed and online consultations had not been a good substitute. The report also pointed to the fact that the number of children/young people at risk had increased during the pandemic.

In its second report in late April (Coordination group 2, 2020), the group pointed out that although the pandemic was by then under control, many public and private institutions still had a lot of precautionary measures in place – more than necessary in their opinion. In addition, many public and private institutions were still urging people to work from home, which had negative effects on vulnerable groups. The group therefore recommended that the relevant institutions re-open and their staff return to normal work in the office. It also recommended carrying out risk evaluations in social and health institutions that were still partially closed and said that cross-institutional contact needed to increase again if vulnerable groups were to be helped. The implementation of these recommendations was slow and even more challenging during the second and third waves.

The Norwegian Institute of Public Health states in a broad meta-study, based on a world-wide set of research articles that: ‘So far in the COVID-19 pandemic there has been almost no research-based evidence of health-related effects of interventions aimed at supporting socially or economically vulnerable groups exposed to the COVID-19 pandemic or transmission control measures’ (NIPH, 2020, 27). Even though this study is very broad and does not specifically focus on the type of social policy responses examined here, it could be indicative.

During the pandemic, the health system as a whole has been geared towards treating COVID-19 patients, among whom old people have been overrepresented. However, one also needs to ask how elderly people have fared in general during the pandemic, or more precisely, how have the social aspects of their lives been managed (Armitage & Nellums, 2020)?

First of all, the authorities’ call to protect elderly people, seems to have been taken ‘too’ seriously by most families, meaning that children and grandchildren cut off physical contact with their parents/grandparents for a year. This was a problematic side-effect of the social distancing policy, which resulted in a general scaremongering climate (Christensen & Lægreid, 2020b). The result was that old people became socially isolated in their homes. This was a special challenge in a culture where old people typically live apart from the rest of the family. The social isolation of the elderly was aggravated further by the fact that public health and social services reduced their services in old people’s homes because of fear of infection. Many of these problems resurfaced in the second and third waves of the pandemic in the fall of 2020. Some geriatricians pointed out that no one had asked the elderly what they wanted during the pandemic. They said that many old people would prefer to have had continuous physical contact with their families and die some months earlier than be socially isolated from them, which they saw as cruel.^2^

Second, Norway has a long tradition of accommodating frail elderly people who can no longer look after themselves in nursing homes or assisted care institutions. This amounts to around 12–13% of the population over eighty. During the first two waves of the pandemic, the official policy was to prohibit relatives from having physical contact with old people, because of the risk that the virus would spread through nursing homes. Family members were allowed to bring gifts, wave to their relatives through the windows or talk to them through open windows, etc., but many elderly people found this sad and awkward, adding to their anxiety about the pandemic.

As indicated, social isolation during the pandemic was a combined effect of official health and social policy and a kind of meta-anxiety in families. Was it justified as an official policy? In some ways it was: there were good health reasons for protecting old people, who often had several pre-existing conditions and hence a much higher death rate as a result of COVID-19 than the average. It has also been argued that Norway was more successful than Sweden in this respect, since Norway had 789 deaths, of which 60% were in care facilities, while Sweden had around 14,574 deaths, with 70% in care facilities (Pierre, 2020).

The treatment of the elderly in Norway during the pandemic was probably not the government’s finest moment, but it is an example of the dynamics of public decision-making processes. The problem, as defined by the authorities, was to protect elderly people from the pandemic. The health policy solution was to isolate them physically from their families, while the alternative definition of social isolation as a negative effect never became an important issue on the public agenda. Moreover, elderly people do not have strong interest groups to give them a voice.

Looking at the connection between the general social policy response and the treatment of the two vulnerable groups, one can conclude that they were rather loosely coupled. One can say that the social policy response had an economic slant and focused on keeping people employed, which indirectly at least may have helped young people, but vulnerable people’s lack of schooling and services in special institutions were defocused. The situation of old people in nursing homes and home care was a side-issue in the social policy response, and they became socially isolated for a long period.

## Discussion

First, how have social concerns been prioritized in the response to COVID-19 in Norway relative to health and economic concerns? Why have vulnerable social groups had to bear heavy burdens in the pandemic – in other countries as well as in Norway (cf. Gaynor & Wilson, 2020; Wright & Merritt, 2020)? The process of deciding on priorities has been dominated by the central political and administrative leadership, in collaboration with expert institutions in a typical top-down process (Christensen & Lægreid, 2020a). In balancing different considerations, the most important criteria for the decisions were the precautionary principle, which is rather unspecific, and major health concerns, including avoiding deaths and serious disease and retaining enough healthcare capacity (Christensen & Lægreid, 2020b). The economic effects of the pandemic were acknowledged, but given a lower priority than health concerns, which was understandable given Norway’s high level of affluence and its willingness to introduce various compensatory measures. The social policy measures overall received much less focus from the central leadership and were rather loosely coupled to vulnerable groups.

The government measures taken to address the negative social effects of COVID-19 were mostly economic ones, reflecting Norway’s solid economic position (NOGOV, 2020a). The indirect ones were connected to large economic support packages to the private sector, while the sector-specific ones were primarily related to income support and job security. The main rationale for this was that by giving businesses and the self-employed a lot of economic support and supporting other sectors of the population via a package of individual measures through the welfare administration, lay-offs could either be avoided or the problems that they caused could be mitigated and hence some degree of normality restored to the lives of families.

How could this general and economically oriented policy help vulnerable groups with diverse health and social concerns, whether young or old (cf. Martin-Howard & Farmbry, 2020)? Regarding vulnerable young people, income support and job security were obviously of indirect relevance since they had positive effects on families, but they did very little to compensate for the loss of daily social networks and the support offered by school and special institutions, etc. Old people got much more attention, but the policy towards this group failed in two ways: a lot of old people in nursing homes died and they suffered from social isolation, which was the result of a paternalistic attitude on the part of the government.

Going back to our perspectives, the *instrumental-structural perspective* can explain much of how the government decided to combat COVID-19. Concerning the control aspect, as already mentioned the process was top-down, which is quite common in major crises (cf. Kettl, 2004). The prime minister and the lead ministers were the most important actors. They consulted expert institutions, but in many ways they had the upper hand in that relationship.

Concerning the definition aspect, meaning how to define goals, problems and solutions (cf. March & Olsen, 1976), the government decided early on to focus on health issues and the capacity of the health system, which they feared would become overwhelmed. The government had broad support in setting this priority (Christensen & Lægreid, 2020b). Regarding solutions or measures, the government claimed that its policy was based on advice from expert institutions. In reality, however, it used the precautionary principle to ‘exaggerate’ and furthered a kind of ‘more is best’ scenario, which deviated from the NIPH, which recommended softer measures, such as keeping schools open. Had kindergartens/schools and social/ psychiatric institutions for children/young people been kept open, vulnerable groups would have retained more of their services and support.

Regarding the government’s lack of focus on social effects, especially in the first regulatory phase, one instrumental cause of this was that these effects had weak institutional support internally in the public apparatus; one indicator of this was that the minister in charge very seldom participated in the daily press conferences (Christensen & Lægreid, 2020b). Moreover, the relevant societal interest groups were not strong enough to push their problems onto the agenda. The policy focus on income support and job security gave social policy an economic slant and defocused vulnerable groups.

Seen from a *cultural-institutional perspective*, the response to COVID-19 was very much in keeping with national tradition, featuring a hierarchically strong government with a tripartite collaboration with employers and employees and more focus on the labor market than on social problems (cf. Christensen, 2003). Thus, the actor patterns and definitions of problems and solutions were rather similar to those in non-crisis situations, where weak groups are defocused and their problems increase. Even though the pandemic was a great shock to the country, it did not result in a new trajectory (cf. Kingdon, 1984). Citizens’ acceptance of the draconian measures taken was in line with what Easton (1965) labels ‘diffuse support’ for the system and for its institutions and leaders, thus reinforcing governance capacity and legitimacy (Christensen et al., 2016). During the pandemic, *trust* in government – as well as in the PM, the health authorities, parliament and national/local politicians – increased from an already high level (Medborgerpanelet 2020).

Regarding a *symbolic perspective*, political leaders used a whole arsenal of symbols for their crisis management (Christensen & Lægreid, 2020b). One was the rather paternalistic attitude of ‘we know what is best for you’, which asserted that if people did what the government told them to do, their lives would eventually return to normal, which turned out to be a long shot. These symbols worked well for the government, even though the rewards were continually postponed, and they were further supported by symbols related to basing the measures on expert advice, which in reality was only partly true. Another major, more or less explicit symbol that came in several versions went along the lines of – ‘Norway is so rich that we can use a lot of money to fight the effects of the pandemic’. This symbol was also well received, even though some groups thought the money was unevenly allocated and had not benefited them.

The symbols related to protecting vulnerable groups were also well received, but were more problematic (Ursin et al., 2020). These were rather unspecific, often along the lines of ‘we care so much about you’ and represented a lot of ‘double talk’ from the government (cf. Brunsson, 1989). The general measures that focused directly on the social effects of the pandemic had an economic profile while the more specific measures regarding vulnerable groups were either too few or else problematic; however, the government did not encounter much opposition to its failure to focus on vulnerable groups.

## Conclusion

The main lesson of this study is that even in an affluent country like Norway with a well-developed welfare state, the government did not do a good job of protecting vulnerable groups from the virus and was sluggish in its response to the social impact of the pandemic (cf. Yates & Dickinson, 2021). This impact was partly a side-effect of the strong measures to stop the COVID-19 surge, especially the periodic closure of kindergartens/schools and the social isolation of old people. Adding to this, a path-dependent culture of deemphasizing the needs of vulnerable groups continued into the pandemic. These groups were also unable to resist the barrage of paternalistic and scaremongering symbols from the government (Christensen & Lægreid, 2020b). The two vulnerable groups studied had few support groups and were largely forgotten when the government designed its social policy response. The specific social policy measures connected to job security and income support did not have much positive effects on these vulnerable groups.

What can one learn from this study? One insight is that vulnerable groups, in this study meaning unprivileged children and youths and certain old people, are losers in the handling of the pandemic, like they are more generally, in a comparative perspective (Chung & Yi, 2021; Gaynor & Wilson, 2020). This means that, as one may have expected in a welfare state like Norway, there is no extra focus or resources directed towards these groups, in any compensatory efforts, since these group face negative social and psychological effects of the pandemic (Ursin et al., 2020; Øverlien, 2020). Second, this is very much reflecting another well-known insight, that vulnerable groups lack social capital and have problems organizing (Armingeon & Schädel, 2019), and therefore influence public decisions less, as reflected in the handling of the pandemic in Norway, i.e. they were not included and listen to.

## Footnotes

1 In December 2020, experts claimed that during the first nine months of the pandemic around 15,000 such ‘single decisions’ taken in kindergartens and schools were not implemented because of lack of resources and reluctance by personnel to engage with the children because of the risk of infection.

2 Advice and priorities from the Norwegian Geriatrics Association. March 30, 2020.
